# Supplementary material for: Baseline and Kinetic Circulating Tumor Cell Counts Are Prognostic Factors in a Prospective Study of Metastatic Colorectal Cancer
Source: Diagnostics (Basel). 2021 Mar 12;11(3):502. doi: 10.3390/diagnostics11030502 (PMC7999095; doi:10.3390/diagnostics11030502)
Supplement: Supplementary file 1 [file diagnostics-11-00502-s001.pdf]

Supplementary files

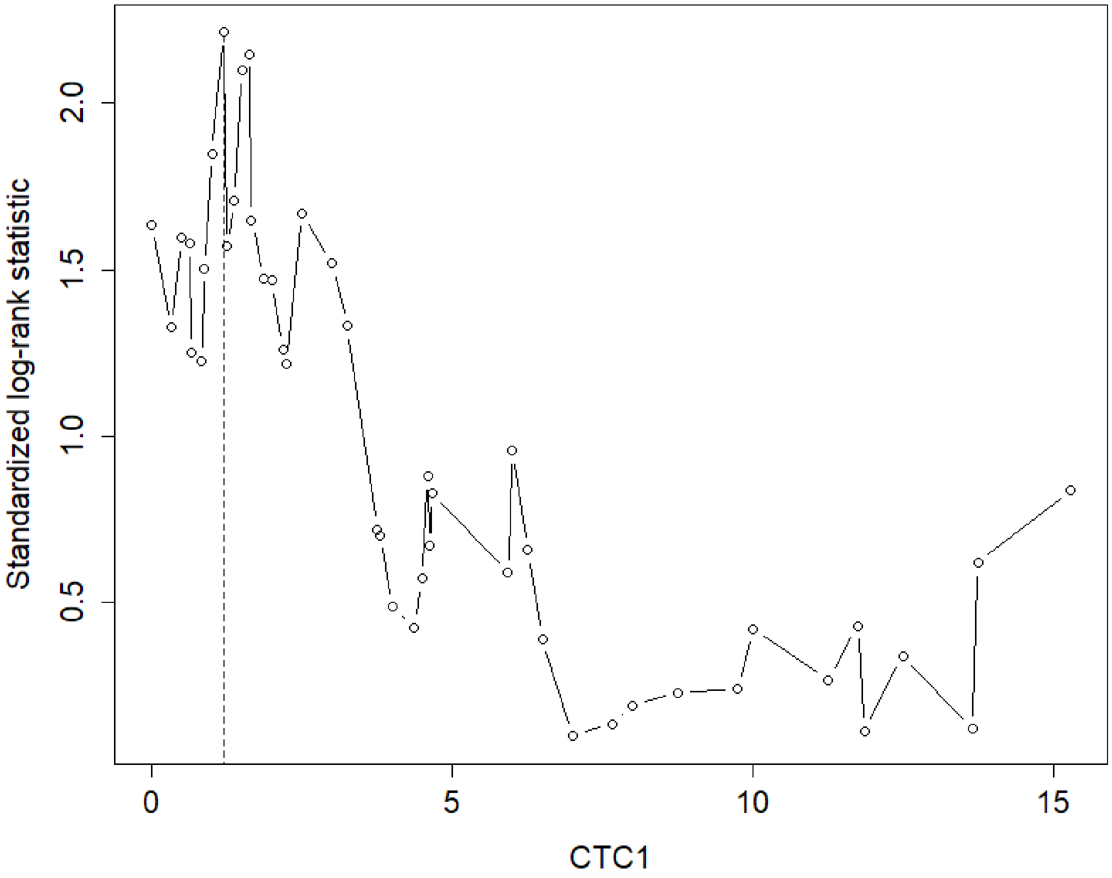

Supplementary Figure S1. Lausen and Schumacher method to determine the best cut-off point for CTC1

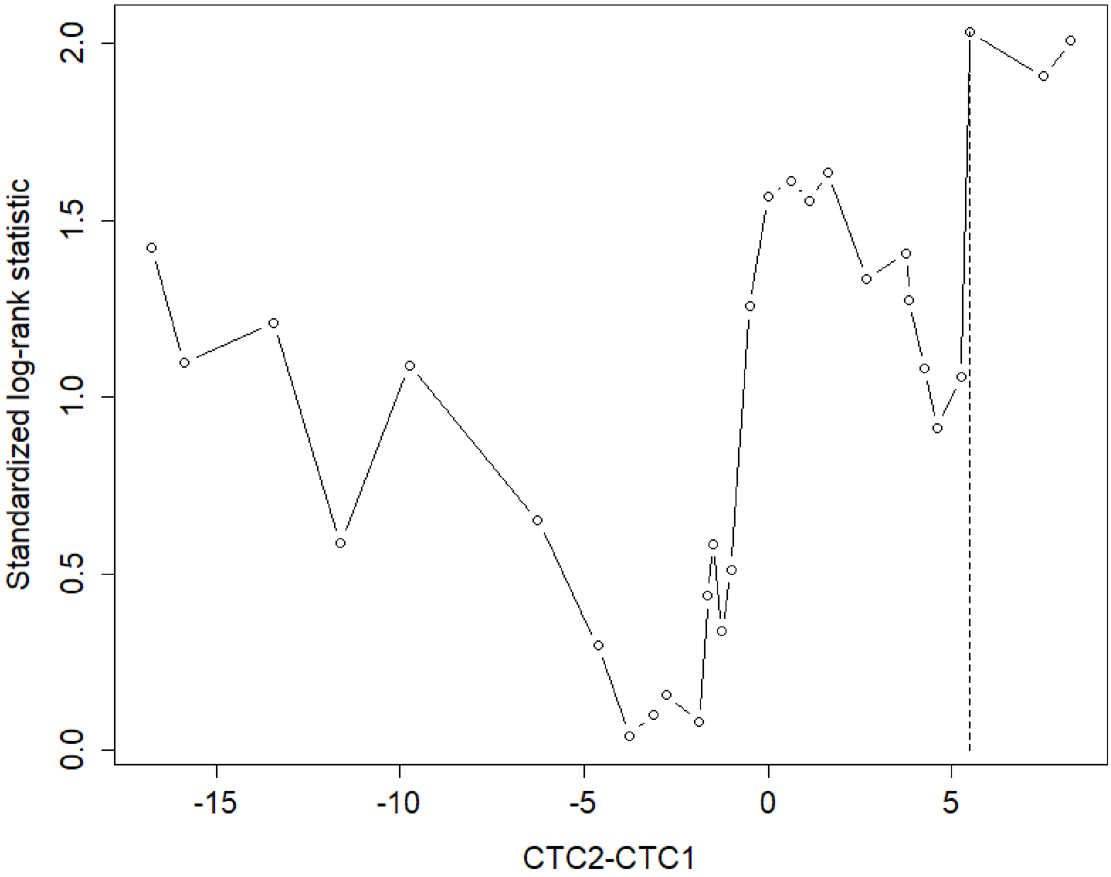

Supplementary Figure S2. Lausen and Schumacher method to determine the best cut-off point for delta CTC (CTC2-CTC1)
